# BUILDING AN ECONOMY THAT SUPPORTS WORKING WOMEN TO THRIVE

**DOI:** 10.1093/geroni/igad104.1919

**Published:** 2023-12-21

**Authors:** Heather McCulloch

**Affiliations:** The Aspen Institute, San Francisco, California, United States

## Abstract

Building on previous research about working women’s financial wellbeing, a provocative question arises: “What would the economy look like if were designed to work for women?” To answer this question, Heather McCulloch, entrepreneur in residence with the Aspen Institute Financial Security Program, conducted a translational research initiative called Women in the Economy. Intended to take a deep dive into the economic lives of working women across the U.S. who face one or more barriers, in addition to gender, to building their economic security, the project included in-depth interviews with 127 women across urban, suburban, and rural communities in 30 states and D.C. Participants were asked about the economic challenges they face, what they see as the most promising solutions, and their vision for a gender-equitable economy that enables women to strive, thrive, and reach their full potential. Findings revealed that working woman are struggling to make ends meet; they face a financial penalty - a loss of income and savings - due to caregiving; and they are not receiving the education they need to successfully navigate economic and financial systems. Building on qualitative results, a national survey (n=1224) was deployed to understand the scope of emergent topics. Among key findings are that four out of five respondents reported feeling economically insecure and only one in five reported feeling confident they would live comfortably in retirement. Together, findings based on the wisdom, voices, and experiences of working women point to policy, program, and market-based solutions that can build a gender-equitable society.

